# Shotgun Metagenomic Survey of Maize Soil Rhizobiome

**DOI:** 10.1128/MRA.00860-20

**Published:** 2020-09-24

**Authors:** Olubukola Oluranti Babalola, Saheed Adekunle Akinola, Ayansina Segun Ayangbenro

**Affiliations:** a Food Security and Safety Niche, Faculty of Natural and Agricultural Sciences, North-West University, Mmabatho, South Africa; Georgia Institute of Technology

## Abstract

The plant soil rhizobiome induces critical functions in the plant proximal environment. Linkages between soil microbiota and primary functional attributes are underexplored. Here, we present the metagenomes of maize soil rhizosphere organisms with functional diversity associated with farms at two different municipalities in North West and Gauteng provinces of South Africa. We describe a plenteous and diverse microbial community.

## ANNOUNCEMENT

South Africa is a semiarid area that is ranked as one of the world’s hot spots inhabited by essential soil organisms, with diverse functional traits that could be useful as biofertilizers. Lichtenburg is a town situated at the center of Ditsobotla municipality (North West Province, South Africa), while Randfontein is a city known for gold mining that is located in the West Rand (Gauteng Province, South Africa) (1). Both areas are covered by grassland biome and are known for substantial maize production. North West Province is a cornerstone of commercial maize production, having produced 40% of the South African annual maize production, with a significant contribution to the economic growth of South Africa (2). The productivity level of the study area may be a result of the plant growth-beneficial microorganisms harbored by South African soil. The appropriate method for gaining adequate information about the microbial structure is through metagenomics. Therefore, this study aimed to assess the functional attributes of the maize soil rhizobiome associated with this environment.

Soil samples were collected in triplicate from the rhizosphere of maize from two different farms in North West Province in South Africa. Samples from farms located at Lichtenburg (25°59′40.4ʺS, 26°31′44.5ʺE) (sample Ls) and Randfontein (26°11′52.0ʺS, 27°33′18.3ʺE) (sample Rs) were collected at a radius of 3 cm from the plant, at a depth of 20 cm, with an area of 2 by 4 m^2^; the surrounding soils were collected from an adjacent area 10 m from each sampling site (3). The collected samples were transported to the laboratory in a cooler box filled with ice and were stored at −20°C for 1 week. Using the DNeasy PowerMax soil kit, DNA was extracted from 5 g of each soil sample following the manufacturer’s instructions. The libraries were prepared using the Nextera DNA Flex library preparation kit. Twenty to 50 ng DNA was used to prepare the libraries. The samples underwent fragmentation and addition of adapter sequences. The final concentrations of the libraries were measured using the Qubit double-stranded DNA (dsDNA) HS assay kit (Life Technologies), and the average DNA fragment lengths were determined using a 2100 Bioanalyzer (Agilent Technologies). The libraries were pooled, diluted to 0.6 nM, and subjected to paired-end sequencing for 300 cycles using the NovaSeq system (Illumina). The downstream analysis of the reads was performed using the default settings of the Metagenomic Rapid Annotations using Subsystems Technology (MG-RAST) server v4.0.3 (4) (Table 1). With the MG-RAST server, the quality control of raw reads was performed using SolexaQA to trim low-quality reads and to dereplicate the metagenomic data. Assessment of the sample sequencing error based on artificial duplicate read measuring was achieved using duplicate read inferred sequencing error estimation (DRISEE). Also, the pipeline adopts the Bowtie aligner to screen the reads for unwanted genomes related to model organisms such as mouse, human, cow, and other animals (4). Using the same pipeline, the BLAST-like alignment tool (BLAT) algorithm was used to annotate the sequences (5) against the M5NR database (6), which provides nonredundant incorporation of different databases.

**TABLE 1:** Basic analytical statistics, quality information, and project accession numbers from NCBI and MG-RAST databases

Sample	No. of raw sequence reads	NCBI BioProject accession no.	No. of reads that passed quality control	No. of known proteins predicted
Ls	19,276,118	PRJNA645371	17,596,177	15,344,917
Rs	14,928,201	PRJNA645385	13,823,192	12,427,664

Taxonomic description at the domain level showed that 98.3 to 98.7%, 0.7 to 1.0%, and 0.5 to 0.6% of the mean read values were assigned to bacteria, eukaryota, and archaea, respectively. Bacterial phyla such as *Proteobacteria* (39.1 to 42.0%) and *Actinobacteria* (35.6 to 36.8%) were the most abundant; others such as *Acidobacteria* (3.9 to 5.5%), *Bacteroidetes* (3.4 to 3.9%), *Deltaproteobacteria* (3.11 to 4.44%), *Solibacteres* (1.74 to 3.81%), *Gammaproteobacteria* (2.93 to 3.70%), and *Gemmatimonadetes* (2.3 to 3.8%) were also significant. Fungal and archaeal reads were assigned mainly to *Ascomycota* and *Basidiomycota* and to *Thaumarchaeota* and *Euryarchaeota*, respectively, but with <1% abundance.

Functional annotation revealed that reads mapped using SEED subsystems were attributed to carbohydrates (14.1 to 14.3%), cluster-based systems (12.8 to 12.9%), amino acids and derivatives (10.4 to 10.7%), protein metabolism (8.1 to 8.3%), stress response (3.0 to 3.1%), etc. The present shotgun metagenomic project reveals the important metabolic prospects of the study area.

### Data availability.

The raw sequences are available under BioProject accession numbers PRJNA645371 (sample Ls) and PRJNA645385 (sample Rs). Quality-filtered and annotated data for each replicate are in the MG-RAST database with accession numbers mgm4898551.3, mgm4898552.3, and mgm4898555.3 (sample Ls) and mgm4898558.3, mgm4898574.3, and mgm4898575.3 (sample Rs).
